# Standardizing Survey Data Collection to Enhance Reproducibility: Development and Comparative Evaluation of the ReproSchema Ecosystem

**DOI:** 10.2196/63343

**Published:** 2025-07-11

**Authors:** Yibei Chen, Dorota Jarecka, Sanu Ann Abraham, Remi Gau, Evan Ng, Daniel M Low, Isaac Bevers, Alistair Johnson, Anisha Keshavan, Arno Klein, Jon Clucas, Zaliqa Rosli, Steven M Hodge, Janosch Linkersdörfer, Hauke Bartsch, Samir Das, Damien Fair, David Kennedy, Satrajit S Ghosh

**Affiliations:** 1 McGovern Institute for Brain Research Massachusetts Institute of Technology Cambridge, MA United States; 2 Flexxbotics Inc Boston, MA United States; 3 Neuro Data Science ORIGAMI Laboratory McConnell Brain Imaging Centre, Faculty of Medicine McGill University Montréal, QC Canada; 4 The Hospital for Sick Children Toronto, ON Canada; 5 BigHat Biosciences San Mateo, CA United States; 6 Child Mind Institute New York, NY United States; 7 McGill Centre for Integrative Neuroscience Montréal Neurological Institute-Hospital Montréal, QC Canada; 8 Department of Psychiatry University of Massachusetts Chan Medical School Worcester, MA United States; 9 Center for Human Development University of California, San Diego San Diego, CA United States; 10 Department of Informatics University of Bergen Bergen Norway; 11 McConnell Brain Imaging Center McGill Centre for Integrative Neuroscience Montréal Neurological Institute, McGill University Montréal, QC Canada; 12 Institute of Child Development University of Minnesota Minneapolis, MN United States; 13 Department of Pediatrics University of Minnesota Minneapolis, MN United States; 14 Masonic Institute for the Developing Brain University of Minnesota Minneapolis, MN United States; 15 Department of Otolaryngology Harvard Medical School Cambridge, MA United States

**Keywords:** reproducibility, survey, data collection, schema, findability, accessibility, interoperability, and reusability, FAIR

## Abstract

**Background:**

Inconsistencies in survey-based (eg, questionnaire) data collection across biomedical, clinical, behavioral, and social sciences pose challenges to research reproducibility. ReproSchema is an ecosystem that standardizes survey design and facilitates reproducible data collection through a schema-centric framework, a library of reusable assessments, and computational tools for validation and conversion. Unlike conventional survey platforms that primarily offer graphical user interface–based survey creation, ReproSchema provides a structured, modular approach for defining and managing survey components, enabling interoperability and adaptability across diverse research settings.

**Objective:**

This study examines ReproSchema’s role in enhancing research reproducibility and reliability. We introduce its conceptual and practical foundations, compare it against 12 platforms to assess its effectiveness in addressing inconsistencies in data collection, and demonstrate its application through 3 use cases: standardizing required mental health survey common data elements, tracking changes in longitudinal data collection, and creating interactive checklists for neuroimaging research.

**Methods:**

We describe ReproSchema’s core components, including its schema-based design; reusable assessment library with >90 assessments; and tools to validate data, convert survey formats (eg, REDCap [Research Electronic Data Capture] and Fast Healthcare Interoperability Resources), and build protocols. We compared 12 platforms—Center for Expanded Data Annotation and Retrieval, formr, KoboToolbox, Longitudinal Online Research and Imaging System, MindLogger, OpenClinica, Pavlovia, PsyToolkit, Qualtrics, REDCap, SurveyCTO, and SurveyMonkey—against 14 findability, accessibility, interoperability, and reusability (FAIR) principles and assessed their support of 8 survey functionalities (eg, multilingual support and automated scoring). Finally, we applied ReproSchema to 3 use cases—NIMH-Minimal, the Adolescent Brain Cognitive Development and HEALthy Brain and Child Development Studies, and the Committee on Best Practices in Data Analysis and Sharing Checklist—to illustrate ReproSchema’s versatility.

**Results:**

ReproSchema provides a structured framework for standardizing survey-based data collection while ensuring compatibility with existing survey tools. Our comparison results showed that ReproSchema met 14 of 14 FAIR criteria and supported 6 of 8 key survey functionalities: provision of standardized assessments, multilingual support, multimedia integration, data validation, advanced branching logic, and automated scoring. Three use cases illustrating ReproSchema’s flexibility include standardizing essential mental health assessments (NIMH-Minimal), systematically tracking changes in longitudinal studies (Adolescent Brain Cognitive Development and HEALthy Brain and Child Development), and converting a 71-page neuroimaging best practices guide into an interactive checklist (Committee on Best Practices in Data Analysis and Sharing).

**Conclusions:**

ReproSchema enhances reproducibility by structuring survey-based data collection through a structured, schema-driven approach. It integrates version control, manages metadata, and ensures interoperability, maintaining consistency across studies and compatibility with common survey tools. Planned developments, including ontology mappings and semantic search, will broaden its use, supporting transparent, scalable, and reproducible research across disciplines.

## Introduction

### Background

Ensuring reproducibility in biomedical, clinical, behavioral, and social science research is crucial yet challenging, affecting every stage of the research process, from study design to results reporting [1-5]. Core issues include inconsistencies in research protocol implementation within studies over time (eg, across generations of researchers in a laboratory) and across study sites (eg, multisite projects), variable data collection methods, unclear documentation of methodological choices, selective reporting practices, and limited transparency in data and code sharing [6,7]. While methodological diversity is essential for addressing diverse research questions, reproducibility relies on transparent reporting of methodological decisions to minimize researcher degrees of freedom. Efforts to improve reproducibility often focus on data analysis and dissemination, yet inconsistencies in questionnaire-based data collection are frequently overlooked. Such inconsistencies, whether in structured psychological assessments or general-purpose surveys, undermine internal reproducibility in multisite and longitudinal studies, reducing data comparability and introducing systematic biases.

Inconsistencies in survey-based data collection arise from multiple factors, including variability in translations across languages [8,9], differences in how constructs are operationalized [10], selective inclusion of questionnaire components [2,11], variations in clinical diagnosis criteria [12,13], and inconsistencies in versioning across research teams and time points. Even minor modifications, such as alterations in branch logic, response scales, or scoring calculations, can significantly impact data integrity, particularly in longitudinal studies [14].

These discrepancies have profound consequences in both clinical and research contexts. In clinical settings, even slight deviations in assessment methods can lead to divergent patient outcomes, particularly in multicultural environments where assessment translations need proper cultural adaptation. In research, such inconsistencies undermine study integrity and bias conclusions, posing challenges for meta-analyses and large-scale collaborative studies that require harmonized datasets.

Several initiatives have sought to improve research reproducibility. The findability, accessibility, interoperability, and reusability (FAIR) principles [15] provide high-level guidance for data management and sharing, ensuring that research data are well documented, discoverable, and reusable. While FAIR does not directly address study reproducibility, its principles support transparency and consistency in data handling, which are critical for reproducibility efforts. However, these principles primarily focus on postcollection data curation and accessibility, leaving gaps in standardizing survey-based data collection at the source. Similarly, resources such as the Cognitive Atlas [16] and Cognitive Paradigm Ontology [17] have helped standardize terminologies for cognitive research but do not define data elements such as survey questions and allowable responses. The National Institute for Mental Health Data Archive [18] and the National Library of Medicine Common Data Elements [19] initiative promote standardized data elements, yet their implementation remains inconsistent across studies.

Widely used survey platforms such as Qualtrics (Qualtrics International Inc) and REDCap (Research Electronic Data Capture; Vanderbilt University) [20] provide structured tools for data collection but do not inherently enforce assessment standardization, version control, or interoperability across research teams and time points. While these platforms allow researchers to create and distribute surveys, they do not provide mechanisms to systematically track changes or ensure that identical constructs are measured consistently over time. To address these challenges, a schema-driven approach is needed to define and enforce standardized survey structures, ensuring consistency in question formats, response options, and metadata across studies. The Center for Expanded Data Annotation and Retrieval (CEDAR) Metadata Model [21] provides a structured system for biomedical data annotation, but its primary focus is on postdata collection metadata management rather than ensuring consistency during data collection.

Despite these efforts, ensuring consistency in survey-based data collection remains insufficiently addressed, particularly in longitudinal studies and multiteam research projects, where maintaining assessment comparability over time and across sites is critical. Without a structured framework for defining, managing, and versioning questionnaires at the point of data collection, researchers often face time-intensive and error-prone processes of harmonizing disparate datasets during later stages of analysis. A systematic solution for ensuring consistent data collection within a study—across time and research teams—while allowing flexibility for study-specific requirements is necessary to improve research integrity and facilitate large-scale data sharing and reuse.

### Objectives

**Figure 1:**
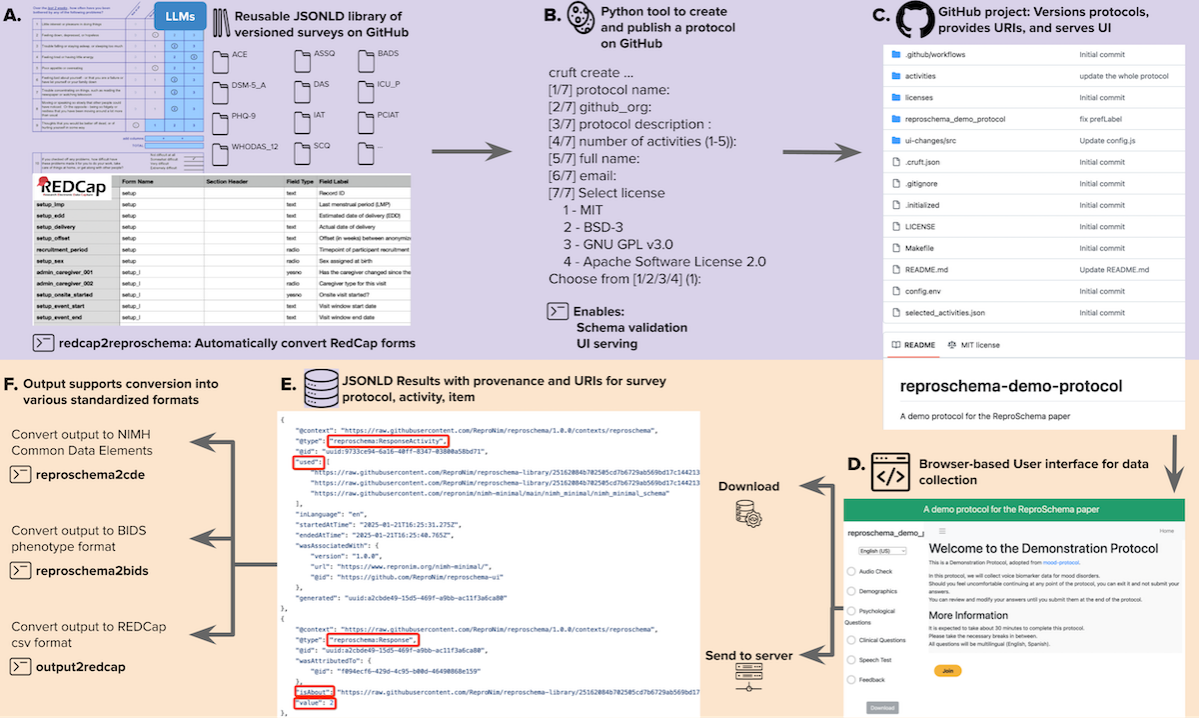
ReproSchema workflow overview. This figure illustrates the 6 core components of the ReproSchema ecosystem. (A) Input sources include Research Electronic Data Capture (REDCap)–formatted CSVs (to be converted using redcap2reproschema), large language model–parsed questionnaire files (eg, PDFs), and a reusable JSON for linked data (JSON-LD) assessment library. (B) Protocols are curated using the reproschema-protocol-cookiecutter tool, which structures metadata, enforces schema validation, and facilitates deployment. (C) GitHub repository versions of each protocol and its components, assigning persistent uniform resource identifiers (URIs) and serving associated web interfaces. (D) The browser-based *reproschema-ui* enables interactive survey deployment and data collection. (E) Survey responses are stored in structured JSON-LD with embedded provenance and links to standardized schema elements. (F) Output data can be converted to various target formats, including National Institute of Mental Health National Data Archive (NDA) Common Data Elements (reproschema2cde), BIDS phenotype format (reproschema2bids), and REDCap CSV (output2redcap), facilitating downstream harmonization and reuse. UI: user interface.

ReproSchema is a schema-driven ecosystem (Figure 1) that integrates a foundational schema with supporting tools to standardize survey-based data collection and facilitate reproducibility. At its core, ReproSchema has a foundational schema that structures and defines assessments, including standardized psychological scales, clinical questionnaires, and general-purpose surveys, by linking each data element (eg, survey response and experimental measurements) with its metadata, such as collection method, timing, and conditions. This structured approach ensures consistency across studies, supports version control, and enhances data comparability and integration.

Beyond the foundational schema, ReproSchema comprises six essential components:

1. A library (reproschema-library) of standardized, reusable assessments, each formatted in JSON-LD [22], providing a structured and versioned resource for common research instruments
2. A Python package (reproschema-py) that supports schema creation, validation, and conversion to formats compatible with existing data collection platforms such as REDCap and the Fast Healthcare Interoperability Resources (FHIR) standard
3. A user interface (UI; *reproschema-ui*) designed for interactive survey deployment, with ongoing development to enhance integration with customized back ends
4. A back-end server (reproschema-backend) for secure survey data submission, using token-based authorization for client registration and data transfer, with support for structured data storage and management
5. A protocol template (reproschema-protocol-cookiecutter) that enables researchers to create and customize research protocols using the standardized assessments and UI
6. A Docker container (*reproschema-server*) that integrates the UI (*reproschema-ui*) and back end (reproschema-backend) to provide a unified platform for deploying protocols and collecting survey data using widely available cloud container services

These components operate both as an integrated system and as stand-alone tools, allowing flexibility in research implementation. Unlike conventional survey platforms that primarily provide graphical user interface–based survey creation, ReproSchema prioritizes schema-based standardization, metadata integration, and interoperability, ensuring that survey elements remain consistent across studies and over time.

Figure 1 presents the ReproSchema workflow, which standardizes survey data collection to enhance research reproducibility and interoperability across studies. The workflow consists of 6 key components. First, ReproSchema supports multiple input formats, including questionnaires in PDF or DOC format (which can be converted to ReproSchema format using large language models, such as Claude 3.7 Sonnet by Anthropic, as demonstrated in Multimedia Appendix 1), existing assessments from the ReproSchema library, and REDCap CSV exports (which can be automatically converted using redcap2reproschema). Second, the *reproschema-protocol-cookiecutter* tool provides a structured, stepwise process for researchers to create and publish a protocol on GitHub, ensuring organized metadata and version control. This tool enables schema validation and UI serving. Third, ReproSchema protocols are stored in GitHub repositories (or other Git-compatible services), where version-controlled uniform resource identifiers (URIs) ensure persistent access to protocols, activities, and assessment items, supporting reproducibility and provenance tracking. Fourth, the *reproschema-ui* provides a browser-based interface for interactive survey deployment, allowing researchers and participants to collect structured data while maintaining schema integrity. Fifth, survey responses are stored in JSON-LD format, with embedded URIs linking each protocol, activity, and item to their respective sources in the ReproSchema library. This structure ensures data provenance, traceability, and semantic interoperability. Sixth, the *reproschema-py* tools facilitate output conversion into various standardized formats, including the National Institute of Mental Health (NIMH) Common Data Elements (reproschema2cde), the Brain Imaging Data Structure phenotype format (reproschema2bids), and REDCap CSV format (output2redcap), ensuring compatibility with existing research workflows.

This paper introduces ReproSchema as a comprehensive framework for addressing inconsistencies in survey-based data collection, thereby enhancing research reproducibility. The Methods section details the conceptual foundation of ReproSchema, describes its components, and presents a comparative analysis against 12 survey platforms. In addition, three research use cases illustrate its applicability: (1) NIMH-Minimal (standardizing required mental health survey common data elements), (2) Adolescent Brain Cognitive Development (ABCD) and HEALthy Brain and Child Development (HBCD) Studies (tracking and managing changes in longitudinal assessments), and (3) Committee on Best Practices in Data Analysis and Sharing (eCOBIDAS; developing an interactive checklist for neuroimaging research best practices). The Results section examines the outcomes of these use cases and highlights how ReproSchema aligns with the FAIR principles. Finally, the Discussion section analyzes the comparative findings, summarizes ReproSchema’s contributions, and outlines current limitations and future directions.

## Methods

### ReproSchema’s Foundation and Implementation

#### Conceptual Framework

#### Overview

A schema, originating in information systems and database design, is a structured framework for organizing data [23]. The concept of schema has been applied to various fields, including web technologies [24,25], where it defines the structure and organization of web-based data. In the context of World Wide Web Consortium protocols [26], schemas ensure web documents adhere to specific formatting and interoperability guidelines.

Beyond web technology, schemas are essential for tracing data provenance, providing transparency and accountability by tracking data’s origins, modification, and lineage [27-29]. In survey-based research, a structured schema maintains consistency and reliability in data collection, especially when assessments vary in complexity and format. A well-defined schema establishes explicit documentation for assessment structure, including question types, response formats, branch logic, and scoring interpretations, thereby ensuring transparency in methodological choices. This approach reduces errors and inconsistencies that arise when researchers implement the same assessment differently, such as variations in scoring methods or selective omissions of items, which can compromise data comparability and reproducibility.

ReproSchema is inspired by the schema-based principles of the CEDAR Metadata Model but has been developed independently to standardize survey-based data collection in biomedical, clinical, behavioral, and social science research. It addresses key challenges such as version control, metadata consistency, and interoperability with existing data collection platforms. This adaptation has resulted in three primary advances.

#### Integration With Established Standards

ReproSchema aligns with schema.org [24] and the Neuroimaging Data Model [30] to enhance data harmonization across studies. For example, schema.org provides a standardized way to describe survey elements, ensuring that metadata (eg, question labels and response formats) remain consistent across platforms. In neuroimaging, Neuroimaging Data Model integration allows seamless linking of behavioral assessments (eg, cognitive tests) with magnetic resonance imaging or functional magnetic resonance imaging data to statistical output [31], enabling researchers to track relationships between questionnaire responses and brain activity. This alignment ensures that datasets remain interoperable, reusable, and compatible with large-scale collaborative studies and meta-analyses.

#### Incorporation of Linked Data Modeling Language

ReproSchema adopts Linked Data Modeling Language (LinkML), an open standard for defining and validating structured data models. LinkML enhances schema expressiveness and validation by enabling explicit data types, relationships, and constraints within survey structures. For example, it allows researchers to specify that a response should be an integer within a defined range (eg, an age field must be between 18 and 99 years) or enforce controlled vocabulary terms (eg, depression severity levels as “mild,” “moderate,” or “severe”). If a questionnaire version changes, such as modifying a Likert scale from a 5-point to a 7-point format, LinkML enables automated detection of such modifications, ensuring that data collected across different versions remains interpretable. This structured validation prevents data entry errors, version mismatches, and inconsistencies that could affect longitudinal analyses or multisite studies.

#### Adaptation to Complex Research Structures

**Figure 2:**
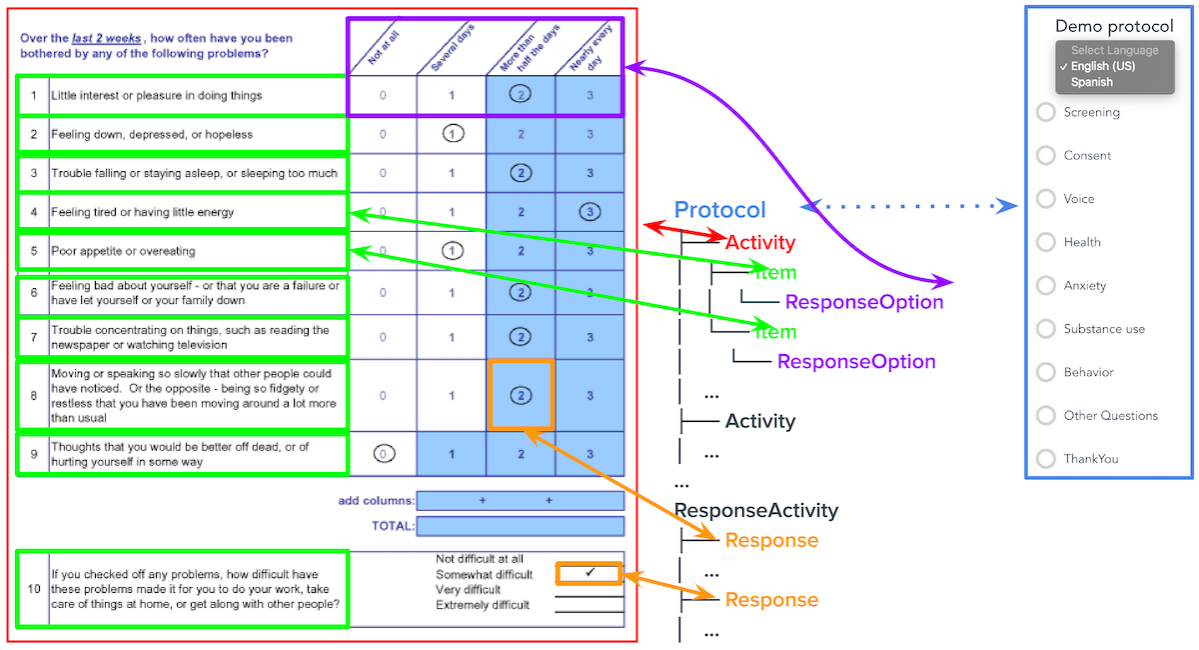
Mapping a research protocol to ReproSchema. This figure illustrates how an assessment is structured and represented in ReproSchema, as well as how it appears in the user interface (UI). The entire assessment (outlined in red) is an activity. Each individual question (green) is an item, and the available answer choices (purple) are ResponseOptions. When a participant selects an answer (orange), that selection is recorded as a response. One protocol can have multiple activities. The right panel demonstrates how different activities within a protocol are organized in the UI, allowing users to navigate between different activities.

ReproSchema uses a nested structure (protocol>activity>item) to represent multilevel research designs (Figure 2). A protocol defines the overall study framework, such as a longitudinal mental health survey or a multisite clinical trial, comprising multiple activities, such as diverse assessments. Each activity can represent a specific assessment, such as the Patient Health Questionnaire-9 for depression screening or a cognitive memory test. An item corresponds to an individual question or measurement, such as “Over the past two weeks, how often have you felt down, depressed, or hopeless?” with a Likert-scale response format. This hierarchical structure allows researchers to reuse standardized assessments, maintain version control, and ensure consistent data formatting (eg, REDCap and FHIR), as explained in the subsequent practical implementation and use cases.

#### Practical Implementation

The implementation of ReproSchema introduces several innovative features aimed at enhancing research efficiency and data integrity.

#### Persistent URI Management via Git

ReproSchema uses Git [32] for robust version control and GitHub, a cloud-based hosting service for Git repositories, for persistent URIs [33] assignment. GitHub serves as a web server to manage URIs for individual schema components, such as protocols, activities, and items. This setup ensures that any schema version remains retrievable through unique Git commits, which function as immutable time stamps for specific code versions. Git is a separate version control system that can be used independently of GitHub, a cloud-based hosting service for Git repositories. Hence, any other Git-based service that provides persistent URLs can also work for *reproschema*. Surveys or questionnaires generated through ReproSchema inherit this version control functionality, enabling researchers to track changes to survey elements (eg, questions and response options) and facilitate study replication or long-term review.

#### Support for Various Question Types

The ReproSchema schema defines a diverse range of question types, including Likert scales, drop-down menus, multiple-choice questions, numerical inputs, audio or video inputs, free-text responses, and more. It also enables conditional visibility (ie, branching logic), where specific items or entire activities become visible based on participant responses.

#### Inclusion of Computable Elements

Beyond defining survey structures, ReproSchema incorporates computable elements to streamline data analysis and enhance automation. The schema allows for predefined calculations on numerical responses, such as computing sums, means, or SDs. For instance, in the DSM-5 Adult Self-Report Cross-Cutting Symptom Measure, ReproSchema can automate the computation of an anxiety subscore by averaging responses from the first 5 questions (eg, “Feeling nervous, anxious, or on edge” and “Not being able to stop or control worrying”).

This automated computation is particularly valuable in large-scale or longitudinal studies, where manual scoring increases the risk of errors and inefficiencies. Moreover, computed values can inform subsequent data collection steps, enabling adaptive survey designs. For example, if a participant’s computed anxiety score exceeds a predefined threshold, additional follow-up assessments could be triggered dynamically. By embedding computational logic within the schema, ReproSchema enables efficient, standardized, and adaptive data collection workflows.

#### Integration of UI Elements

ReproSchema provides a graphical user interface (*reproschema-ui*) for survey execution, enabling participants to complete assessments interactively while ensuring structured data entry. The UI supports a range of input types, including numerical fields, text boxes, multiple-choice selections, sliders, and date pickers. In addition, it accommodates more advanced data capture methods, such as (1) file uploads for submitting scanned documents or images (eg, medical records and task responses), (2) audio checks and recordings for voice-based assessments (eg, speech fluency tasks in cognitive research), and (3) digital consent forms to ensure compliance with ethical requirements in online studies.

The UI is designed to be configurable through JavaScript and is hosted as an open-source GitHub repository, allowing researchers to modify and deploy it according to their study needs. While ReproSchema does not provide built-in survey hosting, researchers can deploy the UI alongside the back end using the Docker-based *reproschema-server*, which integrates both components into a unified deployment. This setup ensures flexibility in different research environments. For example, a researcher studying cognitive decline may deploy a *reproschema-server* instance to collect audio recordings from participants and store them securely for further analysis.

#### Interoperability

ReproSchema’s nested-layer design ensures compatibility with existing data collection and management platforms, enabling seamless conversion and integration with other research workflows. The schema supports bidirectional format conversion, allowing researchers to (1) convert ReproSchema-based assessments to REDCap surveys, ensuring compatibility with widely used clinical research infrastructure, and (2) translate surveys into the FHIR standard [34], facilitating integration with electronic health record systems.

In addition, the modular schema design enables mapping between ReproSchema and other standardized formats. For instance, a research team conducting a multisite study across institutions using different survey tools may use ReproSchema to maintain a centralized, schema-defined questionnaire while allowing site-specific exports to local data collection platforms. We also provide tools to map the data collected through ReproSchema to the REDCap output format and the NIMH submission template.

### Comparison Analysis

#### Overview

This subsection compares ReproSchema’s capabilities to those of other platforms, focusing on the FAIR principles and generic functions found across survey platforms. Through the following selection process and 2 sets of comparison criteria, we highlight the importance of standardized protocols and assessments in improving research outcomes. This comparison evaluates ReproSchema’s role in enhancing the consistency and reliability of research data collection.

#### Identification and Selection of Platforms

On May 20, 2024, we conducted a web search to identify survey data collection platforms commonly used in biomedical, clinical, behavioral, and social science research. We used the keywords “online survey data collection research tool” and “online behavioral experiment data collection research tool” in 2 separate searches, including “behavioral experiment” to capture tools using questionnaires alongside experiments. We then complemented the search list by consulting relevant experts on tools commonly used in biomedicine, clinical trials, and mental health data collection.

Search results were categorized into platform websites and recommendation articles. Platform websites refer to the official sites of the platforms, while recommendation articles include sources listing recommended tools, such as “best data collection tools for research.” Results from these websites and articles were collected to form a comprehensive list. The search results were iterated until no new tools appeared in 10 consecutive results. Results from the 2 searches were then combined to ensure comprehensive coverage. Only tools with at least a web version available were included.

A total of 53 tools were identified: 4 (8%) from the expert panel, 36 (68%) from the “online survey data collection research tool” search, and 13 (25%) from the “online behavioral experiment data collection research tool” search. We filtered those tools based on 2 criteria: research and academia orientation and primary functionality type. Tools were labeled “yes” for research and academia orientation if their websites explicitly indicated use for research purposes or if research institutions were mentioned as primary users. The primary functionality types were categorized as questionnaire focused, experiment and questionnaire, and experiment focused. For example, if a platform only supports using questionnaires in conjunction with an experiment, we label this platform as “experiment focused.” Of the 53 tools, 24 (45%) were identified as research and academia focused. From these, we filtered out 46% (11/24) of the tools that were experiment focused, leaving 54% (13/24) of the tools.

**Figure 3:**
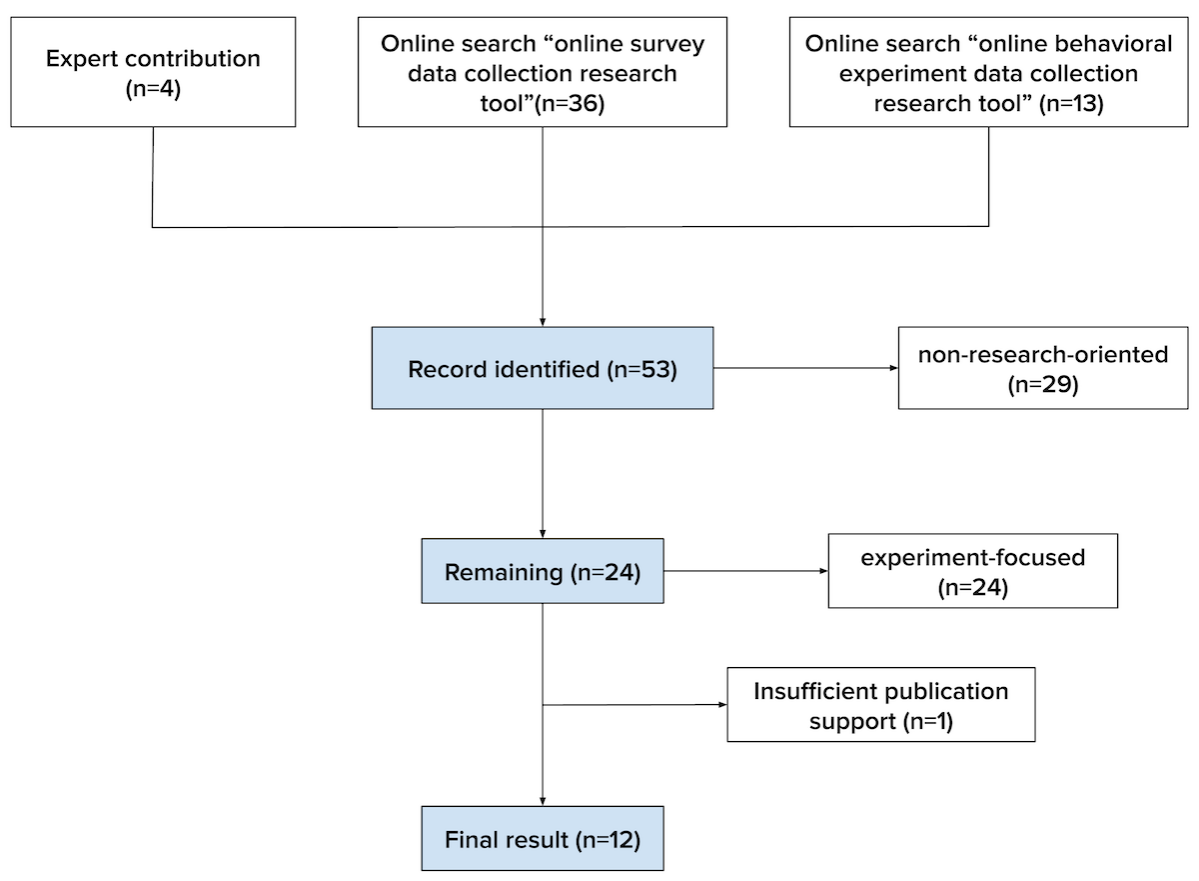
PRISMA (Preferred Reporting Items for Systematic Reviews and Meta-Analyses) flowchart. This figure illustrates the selection process for survey and experiment data collection platforms, narrowing from 53 identified tools to 12 final selections based on research orientation, functionality, and publication support.

We then verified the remaining platforms using Google Scholar, including those with their own publication or that are widely used in publications (ie, at least 10 publications). One platform was excluded due to insufficient publication support. In total, 12 platforms were included in our final comparison. The selection process is illustrated in Figure 3 as a PRISMA (Preferred Reporting Items for Systematic Reviews and Meta-Analyses) chart, with a full list of platforms and selection criteria provided in Multimedia Appendix 2 [20,35-42].

#### Comparison Criteria

We developed 2 sets of comparison criteria to evaluate ReproSchema and the 12 selected tools. The first set focuses on overarching principles for data and metadata management and reusability, while the second set examines practical aspects of tool functionality.

In the first set, we first asked whether the platform had a schema and then evaluated adherence to the FAIR principles (Textbox 1). While originally developed for data stewardship, FAIR principles also contribute to reproducibility by ensuring that research data, including protocols, are well documented and structured to facilitate reuse across studies. The FAIR principles emphasize that data and metadata should be well documented, easily discoverable, accessible under clear conditions, interoperable through shared standards, and reusable through detailed provenance and licensing information.

In the context of survey and questionnaire design and curation before data collection, we interpret “data” as elements within a questionnaire (eg, single questions and response options) and “metadata” as information associated with the questionnaire and its elements during design. In Multimedia Appendix 2, we provided the original FAIR principles and our adapted version side by side to ensure clarity and operationalize these principles for our comparison. Because the FAIR principles serve as general guidelines for scientific data management and our comparison focuses specifically on the survey and questionnaire design phase, some criteria may not be directly applicable in our context. Nonetheless, we listed all criteria for completeness but excluded 1 less relevant standard (R4) from the comparison, as the focus of our work does not involve the relevant standards in the field.

**Adapted findability, accessibility, interoperability, and reusability (FAIR) principles used as the comparison criteria.**

**Findability**

- F1: Does the platform assign unique and persistent identifiers to questionnaires and their elements (eg, questions and response options)?
- F2: Does the platform support metadata to describe questionnaires, including description, version, date created, etc?
- F3: Is it easy to associate metadata with the underlying questionnaire?
- F4: Are questionnaires and their elements indexed in a searchable database or repository?

**Accessibility**

- A1: Can questionnaires and their elements be accessed using standardized protocols (eg, HTTP and Representational State Transfer Application Programming Interface)?
- A2: Are the protocols used for accessing questionnaires open, free, and widely supported?
- A3: Does the platform support secure access mechanisms (eg, OAuth [Open Authorization] and application programming interface keys)?
- A4: Is the metadata describing the questionnaires accessible even if the actual questionnaire’s content is removed or archived?

**Interoperability**

- I1: Are standardized formats used for representing surveys and their elements (eg, JSON and XML)?
- I2: Does the platform use standardized sets of terms and definitions to describe questionnaires and their parts (such as questions and answer choices)?
- I3: Are references to related questionnaires, question items, or external resources included?

**Reusability**

- R1: Detailed metadata: Is detailed metadata provided for questionnaires and their elements, covering all relevant aspects (eg, methodology and intended use)?
- R2: Are clear and accessible use licenses provided for surveys and their elements?
- R3: Is detailed provenance information available for surveys and their elements, including creation and modification history?
- R4: Do surveys and their elements comply with relevant standards in the field? (excluded)

Our second set of criteria (Textbox 2) focuses on general survey platform functions, emphasizing survey design and curation rather than post–data collection procedures. To support consistent evaluation, we anchored the “shared assessments” criterion in widely used mental health instruments (eg, Patient Health Questionnaire-9 and Generalized Anxiety Disorder-7). These assessments offer publicly available, well-defined formats that enable us to determine whether platforms accommodate the reuse of validated instruments. Although this choice draws from mental health research, the intent is not to restrict the scope of evaluation to a single domain. Instead, it reflects a practical approach to assessing platform support for structured and reproducible assessment workflows that extend across research contexts.

Our comparison applied different approaches for the 2 sets of criteria. For the comparison of FAIR principles, we reviewed each platform’s associated publications (specifically publications about the platform itself) and its documentation. This approach was chosen because the FAIR principles relate to design guidelines, which should be reflected in the platform’s foundational design principles.

For the functionality comparison, we relied on platform documentation and created test accounts where applicable to assess the user experience firsthand. In cases where creating a test account was unsuccessful, we referred to Multimedia Appendix 2. This method was chosen because the functionality set is more user oriented, and evaluating it through direct interaction or online documentation provides a practical perspective on the platform’s usability and features.

It is important to note that some platforms (eg, Longitudinal Online Research and Imaging System [LORIS]) provide functions beyond survey design and data collection; however, our comparison only considers the survey-related features. Consequently, our comparison results depend heavily on the platform’s documentation and features available at the time of comparison, which may not always reflect the latest state of the platform.

**The functionality comparison criteria.**

- Shared assessments: whether the platform provides at least 1 of the widely used mental health assessments (eg, Patient Health Questionnaire-9 and Generalized Anxiety Disorder-7) for immediate use
- Multilingual: whether the platform supports multiple languages for assessments
- Multimedia: whether users can directly upload multimedia clips, such as video or audio, into assessments
- Validation: whether the platform offers customizable mechanisms to ensure that entered data meets specific criteria, such as age within a predetermined range
- Logic: whether the platform supports skip or branch logic, allowing the response to a question to determine the subsequent question
- Score: whether users can implement derivative calculations based on questionnaire responses
- Adaptability: whether the platform is optimized for use across various devices, including mobile phones, tablets, and desktops
- Noncode: whether the platform is accessible to researchers without programming skills

### ReproSchema in Research: Overview of Use Cases

This subsection demonstrates ReproSchema’s practical utility in research through 3 use cases of survey design: the NIMH-Minimal initiative [43], the ABCD [44] and HBCD Studies [45], and the eCOBIDAS [46]. These examples highlight ReproSchema’s adaptability and ability to provide tailored solutions for various research needs.

The NIMH-Minimal case illustrates the comprehensive use of ReproSchema in developing standardized data collection protocols. The ABCD and HBCD cases showcase ReproSchema’s utility in standardizing and tracking changes to data elements for longitudinal studies. The eCOBIDAS case demonstrates ReproSchema’s flexibility in various survey types and research contexts.

While all 3 originate from mental health or neuroimaging contexts, their core challenges are broadly applicable. These include tracking historical changes across study waves; managing multisite collaborations; enforcing standardized protocols; and implementing interactive, logic-based assessments. ReproSchema addresses these challenges at the structural level, making the solutions transferable across domains. The Results section further details each use case and the specific problems ReproSchema was designed to solve.

### Ethical Considerations

This study did not involve the collection of identifiable personal data, interaction with human participants, or access to confidential records. As such, ethical review and approval were not required, in accordance with the guidelines of the Massachusetts Institute of Technology’s Committee on the Use of Humans as Experimental Subjects.

## Results

### Overview of ReproSchema Components

The ReproSchema project integrates 5 key components designed to standardize research protocols and enhance consistency across various stages of data collection. These components are briefly described in Table 1, with detailed features and descriptions.

These components, designed to function independently and together, form a comprehensive toolkit catering to various research needs—from creating individual assessments to designing entire study protocols. The ReproSchema project emphasizes flexibility, collaboration, and accessibility, aiming to facilitate a cohesive research process that is both standardized and adaptable to specific research contexts.

Our Multimedia Appendix 2 provides more detailed and up-to-date technical information. The document includes a complete walkthrough to help users set up a new data collection form and describes how to reuse and contribute to the existing library of assessments.

**Table 1:** Each component’s description and key features.

Component	Description	Key features
Foundational schema (*reproschema*)	Provides the structured framework for defining and linking data elements, ensuring consistency, interoperability, and standardization across studies	Provides a standardized framework for structuring questionnaires across diverse research domains, independent of specific topics Standardizes organization, from specific questionnaire items to the entire research protocol
Assessment library (*reproschema-library*)	Provides a collection of standardized questionnaires for various research needs	Covers a wide range of assessments in clinical, cognitive, and behavioral sciences Features version tracking and updating for each questionnaire Open for researchers to add new assessments
Python-based CLI^a^ tool (*reproschema-py*)	Aids in creating, validating, and converting between formats	Standardizes schema creation and validation Provides conversion tools between other formats (eg, REDCap^b^ and FHIR^c^) and ReproSchema formats
UI^d^ (*reproschema-ui*)	Enhances the setup and management of studies, improving data collection	Offers a user-friendly platform for visualizing and adjusting research protocols Flexible use, either stand-alone or integrated with the assessment library
Back end (*reproschema-backend*)	Manages survey data submission and storage, enabling structured, reproducible data collection with token-based authorization	Supports token-based authentication for secure data submission Provides API^e^ end points for integrating with front-end interfaces and external systems
Protocol template (*reproschema-protocol-cookiecutter*)	Offers a user-friendly template for researchers to develop standardized research protocols	Provides a step-by-step guide for protocol development Includes detailed documentation and examples, facilitating implementation
Docker container (*reproschema-server*)	Integrates the UI and back end, streamlining deployment for researchers	Bundles front end and back end into a single deployable unit Simplifies setup and hosting using Docker

^a^CLI: command line interface.

^b^REDCap: Research Electronic Data Capture.

^c^FHIR: Fast Healthcare Interoperability Resources.

^d^UI: user interface.

^e^API: application programming interface.

### Comparison Results

This analysis compares ReproSchema with 12 platforms across 3 distinct research domains: clinical research, general surveys and questionnaires, and online behavioral experiments. The platforms include CEDAR [21], formr [35], KoboToolbox [36], LORIS [37], MindLogger [38], OpenClinica [39], Pavlovia [40], PsyToolkit [41,47], Qualtrics [48], REDCap [20], SurveyCTO [49], and SurveyMonkey [50].

Table 2 provides an overview of each platform and its version at the time of comparison.

**Table 2:** Platform overview and version or and release date.

Platform	Description	Version or release date
CEDAR^a^	A metadata management tool focused on enhancing the annotation and retrieval of biomedical research data, adhering to FAIR^b^ principles	v1.6.0
formr	A survey framework focusing on longitudinal online studies, integrating survey tools with data analysis capabilities	v0.21.0
KoboToolbox	A globally used platform for data collection, management, and visualization in research and social impact initiatives	v2.024.12b
LORIS^c^	A web-based data management system specializing in neuroimaging and behavioral research that can handle longitudinal multisite study data	v25.0.1
MindLogger (rebranded as Curious [51])	A mobile and web platform that collects, manages, and analyzes mental health and behavioral data	v1.25.0
OpenClinica	An electronic data capture platform optimized for clinical trial data management	Community edition (OpenClinica 4)
Pavlovia [52]	A platform for hosting, sharing, and running online experiments and surveys	v2024.1.4 (PsychoPy version)
PsyToolkit	A website for designing, running, and analyzing online experiments and surveys in cognitive and personality psychology	v3.4.6
Qualtrics	A survey tool offering functionalities for academic research, market studies, and customer feedback, with analytics capabilities	May 2024
REDCap^d^	A web application for building and managing online surveys and databases used in clinical and research data collection	v13.8.2
ReproSchema	A system designed for standardizing and sharing research protocols, ensuring reproducibility and consistency across research settings	v1.0.0
SurveyCTO	A data collection platform specialized in mobile data and working in offline settings	v2.81.3
SurveyMonkey	An online survey development software tool that provides survey creation and analysis tools for diverse research needs	May 2024

^a^CEDAR: Center for Expanded Data Annotation and Retrieval.

^b^FAIR: findability, accessibility, interoperability, and reusability.

^c^LORIS: Longitudinal Online Research and Imaging System.

^d^REDCap: Research Electronic Data Capture.

Table 3 presents a comparative analysis of each platform’s performance, evaluated against the adapted FAIR principles. The assessment criteria consisted of 4 categories: findability (F1-F4), accessibility (A1-A4), interoperability (I1-I3), and reusability (R1-R3). The results reveal varying degrees of compliance across the platforms, with some demonstrating stronger adherence to the criteria than others. Table 4 presents each platform’s functional capabilities, highlighting their abilities to support key functions related to survey design and data management.

**Table 3:** Comparison based on the adapted findability, accessibility, interoperability, and reusability (FAIR) principles. The assessment criteria consisted of 4 categories: findability (F1-F4), accessibility (A1-A4), interoperability (I1-I3), and reusability (R1-R3).

Platform	Schema	F1	F2	F3	F4	A1	A2	A3	A4	I1	I2	I3	R1	R2	R3
CEDAR^a^	✓	✓	✓	✓	✓	✓	✓	✓	✓	✓	✓	✓	✓	✓	✓
formr			✓		✓	✓	✓	✓		✓	✓				✓
KoboToolbox			✓		✓	✓	✓	✓		✓	✓				✓
LORIS^b^			✓		✓	✓	✓	✓	✓	✓	✓				✓
MindLogger	✓	✓	✓	✓	✓	✓	✓	✓	✓	✓	✓	✓	✓	✓	✓
OpenClinica			✓		✓	✓	✓	✓		✓	✓				✓
Pavlovia					✓	✓	✓	✓		✓	✓				✓
PsyToolkit					✓	✓	✓	✓		✓	✓				
Qualtrics					✓	✓	✓	✓		✓	✓				✓
REDCap^c^			✓	✓	✓	✓	✓	✓		✓	✓	✓			✓
ReproSchema	✓	✓	✓	✓	✓	✓	✓	✓	✓	✓	✓	✓	✓	✓	✓
SurveyCTO			✓		✓	✓	✓	✓		✓	✓				
SurveyMonkey						✓	✓	✓		✓	✓				

^a^CEDAR: Center for Expanded Data Annotation and Retrieval.

^b^LORIS: Longitudinal Online Research and Imaging System.

^c^REDCap: Research Electronic Data Capture.

**Table 4:** Comparison based on functionality.

Platform	Shared assessment	Multi lingual	Multimedia	Validation	Logic	Score	Adaptability	Noncode
CEDAR^a^	✓		✓					
formr		✓	✓	✓	✓	✓	✓	✓
KoboToolbox			✓	✓	✓	✓	✓	✓
LORIS^b^			✓	✓	✓	✓	✓	✓
MindLogger	✓	✓	✓	✓	✓	✓	✓	✓
OpenClinica		✓	✓	✓	✓	✓	✓	✓
Pavlovia		✓	✓	✓	✓			
PsyToolkit	✓		✓	✓	✓	✓		✓
Qualtrics		✓	✓	✓	✓	✓	✓	✓
REDCap^c^	✓	✓	✓	✓	✓	✓	✓	✓
ReproSchema	✓	✓	✓	✓	✓	✓		
Survey CTO		✓	✓	✓	✓	✓	✓	✓
SurveyMonkey		✓	✓	✓	✓	✓	✓	✓

^a^CEDAR: Center for Expanded Data Annotation and Retrieval.

^b^LORIS: Longitudinal Online Research and Imaging System.

^c^REDCap: Research Electronic Data Capture.

### ReproSchema Use Cases

ReproSchema has been applied in various research settings to address common challenges in survey data collection and management. This section presents 3 applications: NIMH-Minimal, the ABCD and HBCD Studies, and eCOBIDAS. These examples illustrate how ReproSchema provides tailored solutions for standardizing assessments, maintaining version control, and ensuring data consistency across studies (Table 5). By examining these use cases and their associated requirements, researchers can better assess the utility of ReproSchema for their own projects.

**Table 5:** Challenges, requirements, and solutions provided by ReproSchema across use cases.

Challenge	Requirement	Use cases	ReproSchema solutions
Adhering to evolving data collection standards	Version tracking	NIMH^a^-Minimal and ABCD^b^ and HBCD^c^	Git version control with tags for protocols and items
Ensuring uniformity in data collection	Adherence to standardized data collection	NIMH-Minimal	Cookiecutter and UI^d^ creating protocols adhering to guidelines
Customizing assessments based on participant demographics	Tailored data collection for different age groups	NIMH-Minimal	Dynamic UI elements for customizing assessments, such as DSM-5 Cross-Cutting Symptom Measure and WHODAS^e^ 2.0
Maintaining data consistency over time	Data consistency in longitudinal studies	ABCD and HBCD	Systematic tracking and annotation of questionnaire changes
Handling diverse question types	Customizable UI elements	All	Interactive elements such as Likert scales, drop-downs, etc.
Managing complex survey structures	Handling complex survey structures	All	Nested structure (protocol>activity>item)
Integrating with preexisting data collection systems	Interoperability with existing platforms	ABCD and HBCD	Conversion tools such as redcap2reproschema
Overcoming the discouraging length of the original PDF	Interactive format with branching logic	eCOBIDAS^f^	Interactive checklist with branching logic
Ensuring easy access to metadata	Comprehensive metadata accessibility	All	Persistent URIs^g^ for schema components
Supporting multiple languages	Multilingual support	All	Support for multiple languages in UI

^a^NIMH: National Institute of Mental Health.

^b^ABCD: Adolescent Brain Cognitive Development.

^c^HBCD: HEALthy Brain and Child Development.

^d^UI: user interface.

^e^WHODAS: World Health Organization Disability Assessment Schedule.

^f^eCOBIDAS: Committee on Best Practices in Data Analysis and Sharing.

^g^URI: uniform resource identifier.

The NIMH-Minimal project is the NIMH’s recommended data collection instruments for mental health research [43]. These standards specify essential data elements such as age and sex and assessments such as the DSM-5 [53] Cross-Cutting Symptom Measure and World Health Organization Disability Assessment Schedule 2.0 [54] for adults, or age-appropriate equivalents for individuals aged <18 years. ReproSchema ensures compliance with these evolving standards by providing a structured, version-controlled repository for assessments. Tools such as the *reproschema-protocol-cookiecutter* and the *reproschema-library* enable researchers to systematically implement NIMH’s required assessments while maintaining methodological consistency across studies.

ReproSchema outputs responses in JSON-LD format, with each activity stored as a separate file. Both assessments and outputs follow the same schema, ensuring structured, version-controlled, and interoperable data that integrate seamlessly into diverse workflows. To streamline data submission, ReproSchema provides an automated mapping of responses to the National Data Archive (NDA) common data elements format via the reproschema2cde tool. This reduces the burden on researchers while ensuring compliance with NDA standards. Beyond NDA, ReproSchema’s schema-driven structure enables flexible mapping to other formats, enhancing interoperability and simplifying cross-study comparisons.

The ABCD and HBCD Studies, extensive longitudinal studies funded by the National Institutes of Health, investigate the effects of social and environmental factors on child development from pregnancy through adulthood. Both studies initially used REDCap for data collection but faced significant challenges due to frequent questionnaire updates. These updates, such as correcting branching logic errors, fixing typographical mistakes, and modifying response scales, led to data inconsistencies and complications in longitudinal data analysis. ReproSchema addressed these challenges by enabling structured, version-controlled questionnaire development. Each iteration of the questionnaires is systematically tracked using Git tags and annotations, preserving the history of modifications and ensuring that researchers can trace when and why changes were made. The transition from REDCap to ReproSchema improves data consistency and facilitates reuse of standardized data dictionaries, supporting more reliable multisite and longitudinal analyses.

The eCOBIDAS project transformed the extensive 71-page PDF report of the COBIDAS [46,55] into an interactive checklist. The original document detailing best practices for neuroimaging research was often difficult for researchers to navigate due to its length and complexity, which could be overwhelming for researchers. ReproSchema addressed these challenges by converting the report into a dynamic, interactive tool with built-in branching logic, allowing researchers to focus only on the sections relevant to their specific projects. This interactive format reduces information overload and generates automatic, customizable reports that could be directly integrated into manuscripts, enhancing compliance with best practices.

To evaluate ReproSchema’s effectiveness in real-world applications, we conducted an iterative assessment of challenges encountered in survey-based data collection, particularly in longitudinal and multisite studies. We consulted researchers and reviewed documented inconsistencies, identifying common pain points, such as version tracking, metadata accessibility, and interoperability. These challenges were mapped to ReproSchema’s existing features to assess their effectiveness in resolving them. Table 5 summarizes this mapping, demonstrating how ReproSchema provides concrete solutions across different study contexts.

## Discussion

This study presented the ReproSchema ecosystem and evaluated ReproSchema’s role in improving the reproducibility of survey-based data collection by comparing it against 12 widely used platforms. Our findings show that ReproSchema aligns closely with the FAIR principles and provides a structured, version-controlled approach to survey design.

### ReproSchema’s Contribution to Reproducibility

ReproSchema addresses a critical gap in reproducibility efforts by ensuring that survey-based data collection is both standardized and traceable. While initiatives such as the Open Science Framework [56] and the National Institutes of Health reproducibility guidelines [57] emphasize data sharing and transparency, they do not enforce reproducibility at the point of data collection, where inconsistencies often arise.

ReproSchema enhances survey reproducibility in 3 key ways. First, it standardizes data collection by ensuring that all study sites follow consistent assessment protocols, reducing variability in how surveys are designed and administered. Second, it implements robust version control, allowing researchers to systematically track changes to questionnaires over time, ensuring that modifications, such as adjustments to question wording, response scales, or branching logic, are explicitly documented. Finally, it facilitates assessment reuse, reducing the need for post hoc harmonization, which is often error prone and labor intensive.

This structured approach is particularly valuable in longitudinal studies, such as ABCD and HBCD, where even minor inconsistencies in survey instruments across study waves can compromise data reliability and reproducibility. It also supports tracking deliberate updates made to reflect evolving scientific understanding or policy changes. By maintaining survey consistency across time points, ReproSchema supports transparent, reproducible research practices, aligning with broader open science initiatives.

### Comparison Results

#### Overview

The comparison results highlight that while many platforms excel in individual functionalities, they often lack robust metadata management and version control mechanisms. ReproSchema, along with CEDAR and MindLogger, stands out for its ability to preserve questionnaire integrity over time and facilitate cross-study comparability. However, we acknowledge that certain usability challenges remain, particularly for researchers unfamiliar with structured data models.

#### Comparison of Adapted FAIR Principles Across Platforms

#### Findability and Metadata Accessibility

Survey-based research often lacks structured metadata, making it difficult for researchers to locate, reuse, or compare assessments across studies. While many platforms allow users to store questionnaires in searchable databases, they often lack unique, persistent identifiers for individual survey elements, leading to inconsistencies in metadata documentation.

ReproSchema mitigates this issue by assigning structured schemas and unique URIs to every survey component—questions, response options, branching logic, and metadata—ensuring that assessments remain identifiable and retrievable over time. CEDAR and MindLogger adopt similar approaches, reinforcing the role of structured metadata in reproducible research. In contrast, platforms such as formr and Pavlovia, which prioritize experimental flexibility, do not provide the same level of findability.

#### Interoperability and Reusability

Ensuring interoperability is critical for multi-institutional research, yet many platforms store surveys in proprietary or platform-specific formats, restricting their integration with external tools. ReproSchema addresses this limitation by offering bidirectional conversion tools that facilitate seamless translation between ReproSchema and REDCap, as well as between ReproSchema and the FHIR standard. This functionality enables direct integration with clinical and research data management systems, ensuring that assessments remain usable across different platforms while maintaining structured metadata and version control.

Many platforms also struggle with reusability due to limited version control. While REDCap and Qualtrics provide some degree of modification history, they often lack detailed provenance tracking or explicit use licensing. In contrast, ReproSchema’s Git-based version control system allows researchers to track changes systematically and maintain data consistency across study waves, ensuring reproducibility.

#### Comparative Summary of Platform Alignment With FAIR Principles

Among the evaluated platforms, ReproSchema, CEDAR, MindLogger, and LORIS align most closely with FAIR principles, though each has a distinct focus: (1) CEDAR is optimized for biomedical data annotation, (2) MindLogger is tailored for mobile-based mental health assessments, and (3) ReproSchema provides a general-purpose schema for survey-based data collection.

In contrast, platforms such as LORIS, OpenClinica, and REDCap demonstrate partial FAIR alignment, particularly in data management. Platforms such as formr, Pavlovia, and PsyToolkit, developed primarily for psychology research, prioritize experimental flexibility over structured metadata. General-purpose tools such as Qualtrics and SurveyMonkey show variable adherence to FAIR principles, often lacking built-in mechanisms for metadata standardization and reuse.

While FAIR principles are now widely accepted as best practices, many platforms were not originally designed with these principles in mind, explaining the variability in FAIR compliance across platforms.

#### Comparison of Functionality Capabilities Across Platforms

#### Survey Design Features and Usability

Survey tools differ significantly in functionality and intended use. General-purpose platforms such as Qualtrics and SurveyMonkey prioritize ease of use but lack enforced standardization and version control. Research-focused platforms such as REDCap and OpenClinica support structured data collection but provide limited assessment reuse mechanisms. ReproSchema, MindLogger, and formr offer a more comprehensive feature set, including multilingual support, multimedia integration, validation tools, and adaptive branching logic.

#### Mobile Optimization

Unlike MindLogger and Survey CTO, which provide dedicated mobile apps, ReproSchema’s data collection component operates as a web-based platform, ensuring accessibility via mobile browsers. While this ensures device compatibility, certain mobile-native optimizations, such as offline data collection, remain underdeveloped. Expanding support for offline functionality could further enhance ReproSchema’s applicability to mobile health and field-based research, where real-time mobile data collection is essential. While we evaluated skip and branch logic, we recognize that some platforms also support piping, which integrates prior responses into later questions. Though not separately assessed, this is an important feature to consider in future evaluations and schema improvements.

#### Technical Accessibility

One limitation of schema-based platforms is the requirement for programming knowledge. While SurveyMonkey and Qualtrics provide no-code survey builders, ReproSchema currently requires familiarity with structured data models. However, tools such as *reproschema-ui* and *reproschema-protocol-cookiecutter* help lower this barrier, and future development of graphical interfaces and use of artificial intelligence technologies could further improve usability for nontechnical users.

It is important to note that a lack of a feature in our comparison does not necessarily mean its absence—some functionalities may exist but were not explicitly represented in platform documentation. This analysis does not rank platforms but rather highlights trade-offs and strengths, helping researchers select tools suited to their study needs.

### Limitations and Future Directions

While ReproSchema enhances survey-based reproducibility, several areas require further development to improve its accessibility and functionality. Integrating semantic search capabilities into *reproschema-library* could enable researchers to more efficiently identify relevant assessments, streamlining study design and data collection. In addition, ontology mapping, such as implementing the Simple Standard for Sharing Ontological Mappings [58], would improve interoperability with external research standards, allowing for more seamless integration with existing data repositories and standards.

Although ReproSchema’s Git-based version control ensures transparency and traceability, reliance on GitHub may pose accessibility challenges for researchers unfamiliar with version control systems. Exploring alternative or supplementary version control mechanisms could lower this barrier and make the platform more widely usable. Furthermore, expanding integration with participant recruitment platforms, such as Prolific or Mechanical Turk [59-63], could facilitate diverse and large-scale data collection, particularly for behavioral and social science studies. Finally, enhancing documentation and training resources and providing artificial intelligence–based tools would make ReproSchema more accessible to researchers unfamiliar with schema-based approaches, ultimately promoting broader adoption across disciplines.

### Conclusions

ReproSchema provides a structured, metadata-driven approach to survey-based research, ensuring consistency, interoperability, and version control in data collection. By integrating FAIR principles, version tracking mechanisms, and open collaboration, it addresses key barriers to reproducibility in survey research, offering a scalable solution for studies that require standardized, transparent assessment protocols.

Beyond its immediate applications, ReproSchema represents a shift in how survey instruments are designed, maintained, and shared. Its schema-based structure facilitates harmonized data collection across diverse research disciplines, making it a valuable resource for multisite collaborations, longitudinal studies, and cross-disciplinary data integration. As the scientific community continues to emphasize open and reproducible research, tools such as ReproSchema will be crucial for reducing inconsistencies in data collection, especially for multisite and longitudinal collaborative projects.

Future efforts will focus on expanding usability and adoption, particularly by improving graphical interfaces, enhancing interoperability with existing research infrastructures, and lowering technical barriers for nonprogrammers. By addressing these challenges, ReproSchema has the potential to set a new standard for survey-based research, enabling more transparent, reproducible, and scalable data collection practices across biomedical, behavioral, and social sciences.

## Appendix

Multimedia Appendix 1 Example of using Claude 3.7 Sonnet to convert a PDF questionnaire into ReproSchema format. This demonstrates an automated approach to structuring survey data and highlights ongoing efforts to integrate large language models agents into the ReproSchema workflow.

Multimedia Appendix 2 Supplementary material for tool selection and comparison.

## Abbreviations

ABCD: Adolescent Brain Cognitive Development
CEDAR: Center for Expanded Data Annotation and Retrieval
eCOBIDAS: Committee on Best Practices in Data Analysis and Sharing
FAIR: findability, accessibility, interoperability, and reusability
FHIR: Fast Healthcare Interoperability Resources
HBCD: HEALthy Brain and Child Development
LinkML: Linked Data Modeling Language
LORIS: Longitudinal Online Research and Imaging System
NDA: National Data Archive
NIMH: National Institute of Mental Health
PRISMA: Preferred Reporting Items for Systematic Reviews and Meta-Analyses
REDCap: Research Electronic Data Capture
UI: user interface
URI: uniform resource identifier

## Data Availability

All datasets and code generated or analyzed during this study are publicly available in online repositories, including both ReproSchema framework components (ReproSchema core [64], assessment library [65], Python tools [66], user interface [67], backend server [68], protocol template [69], docker deployment [70], demonstration protocol [71], and documentation [72]) and use case repositories (NIMH-Minimal [73], NIMH-Minimal output mapping to common data element [74], Adolescent Brain Cognitive Development and HEALthy Brain and Child Development studies [75], and Committee on Best Practices in Data Analysis and Sharing checklist [76]).
