# Supplementary material for: Standardizing Survey Data Collection to Enhance Reproducibility: Development and Comparative Evaluation of the ReproSchema Ecosystem
Source: J Med Internet Res. 2025 Jul 11;27:e63343. doi: 10.2196/63343 (PMC12299943; doi:10.2196/63343)
Supplement: Multimedia Appendix 1 [file jmir_v27i1e63343_app1.pdf]

you are familiar with reproschema structure where an activity schema is about a questionnaire, each item schema is a single question. here is an example of an item schema and an activity schema. I'll give you a pdf of a questionnaire, can you give me the first 3 item schemas and an activity schema.

example1: item schema

```
{
 "@context": "https://raw.githubusercontent.com/ReproNim/reproschema/1.0.0-rc4/contexts/generic",
 "@type": "reproschema:Field",
 "@id": "open_response_coping",
 "prefLabel": "Coping",
 "description": "schema to record audio of coping mechanisms",
 "schemaVersion": "1.0.0-rc4",
 "version": "0.0.1",
 "preamble": "Please respond to the following prompt with as much detail as possible.",
 "question": {
  "en": "What behaviors and thoughts have you been having to better cope with issues that give you the most stress? What are you interested in trying to change (new relationships, boundaries, acceptance, employment, education, exercise, beginning therapy, meditation, travel, health)?",
  "es": "¿Qué comportamientos y pensamientos ha tenido para afrontar mejor los problemas que le causan más estrés? ¿Qué le interesa tratar de cambiar (nuevas relaciones, límites, aceptación, empleo, educación, ejercicio, terapia inicial, meditación, viajes, salud)?"
 },
 "ui": {
  "inputType": "audioPassageRecord"
 },
 "responseOptions": {
  "valueType": "schema:AudioObject",
  "minValue": 0,
  "maxValue": 60000
 }
}
```

example2: activity schema

```
{
  "@context": [
    "https://raw.githubusercontent.com/ReproNim/reproschema/1.0.0-rc4/contexts/generic",
    {
      "voice": "https://raw.githubusercontent.com/ReproNim/reproschema-library/43e7afab312596708c0ad4dfd45b69c8904088ae/activities/VoiceTask/items/"
    }
  ],
  "@type": "reproschema:Activity",
  "@id": "speech_schema",
  "prefLabel": "Speech tasks",
  "description": "activity for speech tasks",
  "preamble": "Click the Record button before speaking and then the Stop button once you are done. Use your typical volume and typical pitch.",
  "schemaVersion": "1.0.0-rc4",
  "version": "0.0.1",
  "ui": {
    "addProperties": [
      {
        "variableName": "animal_fluency",
        "isAbout": "voice:animal_fluency"
      },
      {
        "variableName": "say_ah",
        "isAbout": "voice:say_ah"
      },
      {
        "variableName": "rainbow",
        "isAbout": "voice:rainbow_passage_short_normal"
      },
      {
        "variableName": "caterpillar",
        "isAbout": "voice:caterpillar"
      },
      {
        "variableName": "stress_cause",
        "isAbout": "items/open_response_stress_cause"
      },
      {
        "variableName": "stress_cause_describe",
        "isAbout": "items/open_response_stress_cause_describe"
      },
      {
        "variableName": "coping",
        "isAbout": "items/open_response_coping"
      },
      {
        "variableName": "positive",
        "isAbout": "items/open_response_positive"
      },
      {
        "variableName": "describe_image",
        "isAbout": "voice:describe_image"
      }
    ],
    "order": [
      "voice:animal_fluency",
      "voice:say_ah",
      "voice:rainbow_passage_short_normal",
      "voice:caterpillar",
      "items/open_response_stress_cause",
      "items/open_response_stress_cause_describe",
      "items/open_response_coping",
      "items/open_response_positive",
      "voice:describe_image"
    ],
    "shuffle": false,
    "allow": [
      "reproschema:AutoAdvance",
      "reproschema:AllowExport"
    ]
  }
}
```

I'll create the first 3 item schemas and an activity schema for the PHQ-9 questionnaire using the ReproSchema format.

Let me start with the first 3 item schemas:

```json
{
  "@context": "https://raw.githubusercontent.com/ReproNim/reproschema/1.0.0-rc4/co
  "@type": "reproschema:Field",
  "@id": "phq9_1",
  "prefLabel": "Little interest",
  "description": "schema for little interest or pleasure in doing things item of t
  "schemaVersion": "1.0.0-rc4",
  "version": "0.0.1",
  "question": {
    "en": "Little interest or pleasure in doing things"
  },
  "ui": {
    "inputType": "radio"
  },
  "responseOptions": {
    "valueType": "xsd:integer",
    "minValue": 0,
    "maxValue": 3,
    "multipleChoice": false,
    "choices": [
      {
        "name": {
          "en": "Not at all"
        },
        "value": 0
      },
      {
        "name": {
          "en": "Several days"
        },
        "value": 1
      },
      {
        "name": {
          "en": "More than half the days"
        },
        "value": 2
      },
      {
        "name": {
          "en": "Nearly every day"
        },
        "value": 3
      }
    ]
  }
}
```

```json
{
  "@context": "https://raw.githubusercontent.com/ReproNim/reproschema/1.0.0-rc4/co
  "@type": "reproschema:Field",
  "@id": "phq9_2",
  "prefLabel": "Feeling down",
  "description": "schema for feeling down, depressed, or hopeless item of the PHQ-
  "schemaVersion": "1.0.0-rc4",
  "version": "0.0.1",
  "question": {
    "en": "Feeling down, depressed, or hopeless"
  },
  "ui": {
    "inputType": "radio"
  },
  "responseOptions": {
    "valueType": "xsd:integer",
    "minValue": 0,
    "maxValue": 3,
    "multipleChoice": false,
    "choices": [
      {
        "name": {
          "en": "Not at all"
        },
        "value": 0
      },
      {
        "name": {
          "en": "Several days"
        },
        "value": 1
      },
      {
        "name": {
          "en": "More than half the days"
        },
        "value": 2
      },
      {
        "name": {
          "en": "Nearly every day"
        },
        "value": 3
      }
    ]
  }
}
```

```json
{
  "@context": "https://raw.githubusercontent.com/ReproNim/reproschema/1.0.0-rc4/co
  "@type": "reproschema:Field",
  "@id": "phq9_3",
  "prefLabel": "Sleep problems",
  "description": "schema for trouble falling or staying asleep, or sleeping too mu
  "schemaVersion": "1.0.0-rc4",
  "version": "0.0.1",
  "question": {
    "en": "Trouble falling or staying asleep, or sleeping too much"
  },
  "ui": {
    "inputType": "radio"
  },
  "responseOptions": {
    "valueType": "xsd:integer",
    "minValue": 0,
    "maxValue": 3,
    "multipleChoice": false,
    "choices": [
      {
        "name": {
          "en": "Not at all"
        },
        "value": 0
      },
      {
        "name": {
          "en": "Several days"
        },
        "value": 1
      },
      {
        "name": {
          "en": "More than half the days"
        },
        "value": 2
      },
      {
        "name": {
          "en": "Nearly every day"
        },
        "value": 3
      }
    ]
  }
}
```

Now, here's the activity schema for the entire PHQ-9 questionnaire:

```json
{
  "@context": [
    "https://raw.githubusercontent.com/ReproNim/reproschema/1.0.0-rc4/contexts/gen
    {
      "phq": "https://raw.githubusercontent.com/ReproNim/reproschema-library/maste
    }
  ],
  "@type": "reproschema:Activity",
  "@id": "phq9_schema",
  "prefLabel": "Patient Health Questionnaire-9",
  "description": "PHQ-9 assessment for depression screening",
  "preamble": "Over the last 2 weeks, how often have you been bothered by any of t
  "schemaVersion": "1.0.0-rc4",
  "version": "0.0.1",
  "compute": [
    {
      "variableName": "phq9_total_score",
      "jsExpression": "phq9_1 + phq9_2 + phq9_3 + phq9_4 + phq9_5 + phq9_6 + phq9_
    }
  ],
  "ui": {
    "addProperties": [
      {
        "variableName": "phq9_1",
        "isAbout": "phq:phq9_1"
      },
      {
        "variableName": "phq9_2",
        "isAbout": "phq:phq9_2"
      },
      {
        "variableName": "phq9_3",
        "isAbout": "phq:phq9_3"
      },
      {
        "variableName": "phq9_4",
        "isAbout": "phq:phq9_4"
      },
      {
        "variableName": "phq9_5",
        "isAbout": "phq:phq9_5"
      },
      {
        "variableName": "phq9_6",
        "isAbout": "phq:phq9_6"
      },
      {
        "variableName": "phq9_7",
        "isAbout": "phq:phq9_7"
      },
      {
        "variableName": "phq9_8",
        "isAbout": "phq:phq9_8"
      },
      {
        "variableName": "phq9_9",
        "isAbout": "phq:phq9_9"
      },
      {
        "variableName": "phq9_difficulty",
        "isAbout": "phq:phq9_difficulty"
      }
    ],
    "order": [
      "phq:phq9_1",
      "phq:phq9_2",
      "phq:phq9_3",
      "phq:phq9_4",
      "phq:phq9_5",
      "phq:phq9_6",
      "phq:phq9_7",
      "phq:phq9_8",
      "phq:phq9_9",
      "phq:phq9_difficulty"
    ],
    "shuffle": false,
    "allow": [
      "reproschema:AutoAdvance",
      "reproschema:AllowExport"
    ]
  }
}
```

These schemas follow the ReproSchema structure, where the activity schema represents the entire PHQ-9 questionnaire and each item schema represents an individual question. The activity schema references all nine PHQ-9 items plus the difficulty question and includes computation for the total score.
